# Incomplete childhood immunization in Nigeria: a multilevel analysis of individual and contextual factors

**DOI:** 10.1186/s12889-017-4137-7

**Published:** 2017-03-08

**Authors:** Sulaimon T. Adedokun, Olalekan A. Uthman, Victor T. Adekanmbi, Charles S. Wiysonge

**Affiliations:** 1 Warwick-Centre for Applied Health Research and Delivery (WCAHRD), Division of Health Sciences, University of Warwick Medical School, Coventry, UK; 2 Department of Demography and Social Statistics, Obafemi Awolowo University, Ile-Ife, Nigeria; 3 Centre for Evidence-based Health Care, Faculty of Medicine and Health Sciences, Stellenbosch University, Cape Town, South Africa; 4 NIHR Collaboration for Leadership in Applied Health Research and Care, West Midlands (CLAHRC WM), University of Warwick Medical School, Coventry, UK; 5 South African Cochrane Centre, South African Medical Research Council, Cape Town, South Africa

**Keywords:** Immunization, Children, Incomplete, Multilevel, Factors, Contextual, Model

## Abstract

**Background:**

Under-five mortality remains high in sub-Saharan Africa despite global decline. One quarter of these deaths are preventable through interventions such as immunization. The aim of this study was to examine the independent effects of individual-, community- and state-level factors on incomplete childhood immunization in Nigeria, which is one of the 10 countries where most of the incompletely immunised children in the world live.

**Methods:**

The study was based on secondary analyses of cross-sectional data from the 2013 Nigeria Demographic and Health Survey (DHS). Multilevel multivariable logistic regression models were applied to the data on 5,754 children aged 12–23 months who were fully immunized or not (level 1), nested within 896 communities (level 2) from 37 states (level 3).

**Results:**

More than three-quarter of the children (76.3%) were not completely immunized. About 83% of children of young mothers (15–24 years) and 94% of those whose mothers are illiterate did not receive full immunization. In the fully adjusted model, the chances of not being fully immunized reduced for children whose mothers attended antenatal clinic (adjusted odds ratio [aOR] = 0.49; 95% credible interval [CrI] = 0.39–0.60), delivered in health facility (aOR = 0.62; 95% CrI = 0.51–0.74) and lived in urban area (aOR = 0.66; 95% CrI = 0.50–0.82). Children whose mothers had difficulty getting to health facility (aOR = 1.28; 95% CrI = 1.02–1.57) and lived in socioeconomically disadvantaged communities (aOR = 2.93; 95% CrI = 1.60–4.71) and states (aOR = 2.69; 955 CrI =1.37–4.73) were more likely to be incompletely immunized.

**Conclusions:**

This study has revealed that the risk of children being incompletely immunized in Nigeria was influenced by not only individual factors but also community- and state-level factors. Interventions to improve child immunization uptake should take into consideration these contextual characteristics.

## Background

Although the world witnessed a tremendous reduction in child mortality between 1990 and 2015, sub-Saharan Africa (SSA) is still characterised by high under-five deaths. As at 2015, under-five mortality (U5MR) in the region was 83 deaths per 1000 live births [1]. One-quarter of these deaths are preventable through interventions such as immunization. Reports have indicated that the available vaccines today could prevent an estimated 25% deaths of children under the age of 5 [2]. It is in view of this that the World Health Organization (WHO) and United Nations Children’s Fund (UNICEF) developed the Global Immunization Vision and Strategy with the objectives of increasing the number of children being immunized against preventable diseases, incorporating other interventions with immunization and managing immunization programmes based on global interdependence [3].

This effort was also mirrored in Nigeria as the Expanded Programme on Immunization (EPI) was introduced in the country in 1979. The aim of EPI included reduction of vaccine-preventable diseases and improvement of primary health care delivery in different localities. The programme recorded a major success between 1988 and 1990 when diptheria-tetanus-pertussis (DTP) 3 coverage reached 81.5% [4]. A decline, however, set in around late 1990s, but there have been renewed efforts to revitalize the programme in recent years. Through partnership with international organizations, a comprehensive multi-year strategy was embarked upon to sustainably strengthen the EPI [4]. The strategy aimed at truncating the spread of the poliovirus, introducing new vaccines and improving immunization coverage generally, among others. One of the goals of achieving this aim was to ensure that members of the community were aware of the significance of completing the immunization schedule [4]. In May 2012, Nigeria joined other member states of the World Health Assembly to endorse the Global Vaccine Action Plan; an agenda for universal access to immunisation by 2020 [5].

However, the proportion of children that completed the immunization schedule (that is, those who received BCG against tuberculosis, 3 doses of vaccine against DTP, at least 3 doses of vaccine against polio and 1 dose f vaccine against measles) is still very low; 30% in 1990, 13% in 2003 and 23% in 2008 [6–8]. Through previous studies, the low immunization uptake has been attributed to factors such as maternal education, age, occupation, marital status, residence, access to media, fear of side effects, household wealth and place of delivery [9–11]. Most of these studies have concentrated on individual level factors with little attention paid to contextual characteristics. In the present time, emphasis is being placed on the study of social determinants of health in order to have a better understanding of factors influencing people’s health. These social determinants transcend individual characteristics. It has been observed that even studies that considered contextual factors have limited their focus on children with complete immunization status [12, 13], leaving out those who did not complete their immunization schedule. Therefore, the purpose of this article was to develop and test a model of childhood immunization that includes individual-level characteristics along with contextual characteristics defined at the community and state levels in Nigeria.

## Methods

### Study design

This study used data from the Nigeria Demographic and Health Survey (NDHS) 2013 which is a population-based cross-sectional survey.

### Sampling technique

The selection of sample was based on clusters and households and this involved a three-stage sampling technique. Nigeria was divided into strata which consist of all the 36 states and the Federal Capital Territory (FCT). Enumeration areas (EAs) were created in every state for easy access to the respondents. In the first stage, 896 clusters were randomly selected. The second stage involved a random selection of one EA from most of the clusters and this resulted in the selection of 372 EAs from the urban areas and 532 from the rural areas. A total of 45 households were selected from each rural and urban area. Altogether, 40,680 households were sampled for the survey; 23,940 in the rural areas and 16,740 in the urban areas. Complete details of the methods used in the NDHS have been published elsewhere [14].

### Data collection

Data were collected through the use of questionnaires that were administered by conducting face-to-face interviews. Information obtained through this process covered socioeconomic characteristics, reproductive history, prenatal and postnatal care, nutrition, immunization and HIV/AIDS. Information on immunization was collected through vaccination cards and mothers’ verbal reports. Interviewers asked mothers to present the vaccination cards in order to obtain vaccination dates. In the absence of vaccination cards, such mothers were asked to recall the vaccination administered on to their children. Details of the data collection procedure have been published elsewhere [14].

### Outcome variable

We limited our study to children aged 12–23 months as children at this age are expected to have received all the basic doses of immunization. The outcome variable was calculated using 9 doses of 4 vaccines; Bacillus Calmette–Guérin (BCG) (1 dose), Polio (4 doses), DTP (3 doses) and Measles (1 dose) (see Table 1). The DTP-containing vaccine currently used in Nigeria is a pentavalent vaccine that also includes *Haemophilus influenzae* type b and hepatitis B virus antigens. The NDHS applied the WHO recommendations that stipulate that a child is considered fully immunized if he or she received BCG against tuberculosis; 3 doses of vaccine against DTP; at least 3 doses of vaccine against polio and 1 dose of vaccine against measles [14]. The outcome variable was derived from nine variables which represent the doses. Children who received all the nine doses were categorized as fully immunized and those who received less than nine doses were defined as not completely immunized.

**Table 1:** Routine immunization schedule for children in Nigeria

At birth	BCG + Polio 0
6 weeks	DPT 1 + Polio 1
10 weeks	DPT 2 + Polio 2
14 weeks	DPT 3 + Polio 3
9 months	Measles

Source: Federal Ministry of Health, Nigeria [4]

### Independent variables

#### Individual-level factors

The following variables were considered in the study: mother’s age, education, wealth index, marital status, occupation, sex of child, and birth order. Others include size of child at birth, exposure to mass media, antenatal care and place of delivery. Mother’s age was grouped into 15–24, 25–34 and 35+. Education was defined as no education, primary and secondary or higher. Wealth index was originally presented in 5 quintiles by DHS which were derived from the measurements of ownership of household items such as car, radio, television, and dwelling features like toilet facilities, water source and type of roofing/floor. This mode of measurement has been used by the World Bank to categorize households into poverty levels based on principal components analysis [15, 16]. For easy interpretation, we reclassified the weighted scores into three tertiles (poor, middle and rich). Marital status was grouped into never married and ever married. We categorized maternal occupation as not working and working. Sex of child was defined as male and female. Birth order of children was categorized into 1st -3rd order, 4th -6th order and 7th + order. Size of child at birth was categorized into three; large, average and small. Exposure to mass media refers to the frequency of access to newspaper, radio and television. Those who had access to any of the three outlets (for any number of times in a week) were defined as exposed and others were considered never exposed. Antenatal care was dichotomized as attended for women who paid at least one visit to the clinic during pregnancy and never attended for others. Place of delivery was categorized into health facility for women who delivered at either public or private hospital and home for those who delivered elsewhere.

#### Community-level factors

The factors that were considered at the community level included place of residence, difficulty experienced in getting to health facilities, ethnicity diversity index and community socioeconomic status. Place of residence was grouped into urban and rural. Difficulty experienced in getting to health facilities was defined in terms of distance and lack of transportation. This was categorized into having a problem getting to health facilities and not having a problem. Ethnicity diversity index was defined through a formula given as follows

1 $$ \mathrm{Ethnic}\ \mathrm{diversity}\ \mathrm{index}=1-{\displaystyle {\sum}_{\mathrm{i}=1}^{\mathrm{n}}{\left[\frac{x_i\ }{\mathrm{y}}\right]}^2} $$

Where: *x*_*i*_= population of ethnic group i of the area, y = total population of the area, *n =* number of ethnic groups in the area.

The index measures the number of ethnic groups in a locality [17]. The formula calculates scores from 0 to approximately 1 and each index is multiplied by 100 to show the diversity. A higher index score implies better ethnic diversity. While a diversity of 0 indicates the community is dominated by one ethnic group, a 100 diversity index implies such a community is populated by various ethnic groups that are equally represented. Socioeconomic status of the community was computed from the socioeconomic characteristics such as education, occupation and wealth of individuals living in the same community. Through a principal components method, the proportion of individuals who are; uneducated, unemployed and poor was calculated. A standardized score of 0 mean and 1 standard deviation was derived from the proportion. The scores were then grouped into three tertiles (least disadvantaged, tertile 2 and most disadvantaged) with the highest score representing a lower socioeconomic status.

#### State-level factors

State socioeconomic status was arrived at through socioeconomic characteristics of individuals living within the same state. Such characteristics include education, occupation and wealth. Using a principal components method, proportion of individuals in the same state who are; uneducated, unemployed and poor was calculated. A standardized score of 0 mean and 1 standard deviation was derived from the proportion. The scores were then categorized into three tertiles (least disadvantaged, tertile 2 and most disadvantaged) with the highest score representing a lower socioeconomic status.

### Statistical analyses

#### Descriptive analysis

The descriptive analysis was presented by showing the distributions of independent variables by the outcome variable. The distributions were expressed as numbers and percentages.

#### Modelling approaches

We calibrated a three-level binomial logistic regression that had a structure of children with incomplete vaccination or not at level 1 nested within communities at level 2 from a State at level 3. We have applied binomial logistic regression because of the dichotomous nature of the outcome variable. Four models were constructed. The first model is a null model with no independent variables. This model was included to decompose the amount of variance that existed between the community and state levels. Second model contained individual-level variables. The third and fourth were expanded to include community- and state-level variables respectively.

#### Fixed effects (measures of association)

Fixed effects were presented as odds ratios (OR) with their 95% credible intervals (CrI).

#### Random effects (measures of variation)

The results of random effects comprised an intra-cluster correlation (ICC), a variance partition coefficient (VPC) and median odds ratio (MOR). MOR is the unexplained cluster heterogeneity. Details of the methods applied for computing MOR have been published elsewhere [18, 19].

#### Model Fit and specifications

We used the Bayesian Deviance Information Criterion (DIC) to assess the goodness of fit of the model. Variance Inflation Factor (VIF) was applied to test for multicollinearity. All the multilevel modelling operations were executed using MLwiN 2.35 [20] calling Stata Statistical Software for windows version 14 [21] using (runmlwin). Markov Chain Monte Carlo (MCMC) estimation was used for the multilevel logistic regression models [22]. *P* value of < 0.05 was used to define statistical significance.

## Results

### Descriptive statistics

Table 2 presents the descriptive analysis of the respondents’ characteristics. The analysis involved 5,754 children aged 12–23 months (level 1), nested within 896 communities (level 2), from 37 states (level 3) in Nigeria. More than three-quarter of the children (76.3%) were not fully immunized. About 83% of children of young mothers (15–24 years) and 94% of those whose mothers are illiterate did not receive full immunization. While most children from poor households (94.6%) were not fully immunized, about 91% of children whose mothers never had access to mass media did not receive full immunization. Most children with mothers not attending antenatal clinic (91.6%) and mothers who delivered at home (88.5%) were not fully immunized. The preponderance of children from rural area (85.1%) and children whose mothers had problem accessing health facility (71.8%) did not receive full immunization. About 9 in every 10 children who lived in the most economically disadvantaged communities and states were not fully immunized.

**Table 2:** Child immunization status at different levels of independent variables

Variables	Immunization status		Total N (%)	*P* value
	Fully immunized	Not fully immunized		
	N (%)	N (%)		
Individual-level factors	1,361(23.7)	4,393(76.3)	5,754(100)	
Maternal age				
15–24	281(17.4)	1,332(82.6)	1,613(100)	
25–34	769(27.1)	2,073(72.9)	2,842(100)	
35+	311(23.9)	988(76.1)	1,299(100)	<0.0001
Maternal education				
No education	166(6.4)	2,441(93.6)	2,607(100)	
Primary	274(24.6)	842(75.4)	1,116(100)	
Secondary/higher	921(45.3)	1,110(54.7)	2,031(100)	<0.0001
Wealth index				
Poor	103(5.4)	1,815(94.6)	1,918(100)	
Middle	374(19.5)	1,544(80.5)	1,918(100)	
Rich	884(64.1)	1,034(53.9)	1,918(100)	<0.0001
Marital status				
Never married	54(40.3)	80(59.7)	134(100)	
Ever married	1,307(23.3)	4,313(76.7)	5,620(100)	<0.0001
Maternal occupation				
Not working	333(19.0)	1,422(81.0)	1,755(100)	
Working	1,028(25.7)	2,971(74.3)	3,999(100)	<0.0001
Sex of child				
Male	741(24.6)	2,269(75.4)	3,010(100)	
Female	620(22.6)	2,124(77.4)	2,744(100)	0.071
Birth order				
1st -3rd order	843(28.9)	2,074(71.1)	2,917(100)	
4th -6th order	392(20.7)	1,499(79.3)	1,891(100)	
7th + order	126(13.3)	820(86.7)	946(100)	<0.0001
Size of child at birth				
Large	658(25.8)	1,889(74.2)	2,547(100)	
Average	562(24.3)	1,750(75.7)	2,312(100)	
Small	141(15.8)	754(84.2)	895(100)	<0.0001
Exposure to media				
Never exposed	184(9.3)	1,791(90.7)	1,975(100)	
Exposed	1,177(31.1)	2,602(68.9)	3,779(100)	<0.0001
Antenatal care				
Never attended	186(8.4)	2.017(91.6)	2,203(100)	
Attended	1,175(33.1)	2,376(66.9)	3,551(100)	<0.0001
Place of delivery				
Home	405(11.5)	3,123(88.5)	3,528(100)	
Health facility	956(42.9)	1,270(57.1)	2,226(100)	<0.0001
Community-level factors				
Residence				
Rural	572(14.9)	3,260(85.1)	3,832(100)	
Urban	789(41.1)	1,133(58.9)	1,922(100)	<0.0001
Getting to health facility				
Not a problem	1,115(28.2)	2,843(71.8)	3,958(100)	
A problem	246(13.7)	1,550(86.3)	1,796(100)	<0.0001
Ethnicity diversity index, mean(SD)			2.32(2.92)	
Socioeconomic disadvantage				
Tertile 1(least disadvantaged)	854(43.2)	1,121(56.8)	1,975(100)	
Tertile 2	440(23.6)	1,426(76.4)	1,866(100)	
Tertile 3 (most disadvantaged)	67(3.5)	1,846(96.5)	1,913(100)	<0.0001
State-level factors				
Socioeconomic disadvantage				
Tertile 1(least disadvantaged)	772(38.8)	1,216(61.2)	1,988(100)	
Tertile 2	495(24.6)	1,520(75.4)	2,015(100)	
Tertile 3 (most disadvantaged)	94(5.4)	1,657(94.6)	1,751(100)	<0.0001

### Measures of association

Table 3 shows the results of the different models considered in the study. After adjusting for the effects of individual-, community- and state level-factors, the probability of a child not receiving full immunization reduced by 45% for mothers aged 35 and above compared to mothers who are 15–24 years. The odds of a child not being fully immunized reduced as the level of mothers’ education increased. Children of mothers with no education (OR = 2.14; 95% CrI = 1.59–2.86) and primary education (OR = 1.42; CrI = 1.14–1.76) are more likely to be incompletely immunized compared with children of women with secondary or higher education. Children from poor households have higher probability of not being fully immunized as the odds reduced by 52% for children from rich households. Children of higher birth order (7+) and those considered small at birth were 46% and 32% more likely to be incompletely immunized respectively. The chances of not being fully immunized reduced for children whose mothers attended antenatal clinic (OR = 0.49; 95% CrI = 0.39–0.60), delivered in health facility (OR = 0.62; 95% CrI = 0.51–0.74) and lived in urban area (OR = 0.66; 95% CrI = 0.50–0.82). Children whose mothers had difficulty getting to health facility (OR = 1.28; 95% CrI = 1.02–1.57) and lived in socioeconomically disadvantaged communities (OR = 2.93; 95% CrI = 1.60–4.71) and states (OR = 2.69; 955 CrI = 1.37–4.73) are more likely to be incompletely immunized.

**Table 3:** Factors associated with incomplete child immunization identified by multilevel multivariate logistics regression models

Variable	Model 1^a^	Model 2^b^	Model 3^c^	Model 4^d^
	aOR (CrI)	aOR (CrI)	aOR (CrI)	aOR (CrI)
Measure of association				
Individual-level factors				
Maternal age				
15–24		1 (reference)	1 (reference)	1 (reference)
25–34		0.59(0.48–0.72)	0.61(0.50–0.74)	0.63(0,50–0.79)
35+		0.51(0.38–0.67)	0.53(0.40–0.69)	0.55(0.40–0.74)
Maternal education				
No education		2.68(1.99–3.50)	2.12(1.58–2.78)	2.14(1.59–2.86)
Primary		1.413(1.13–1.75)	1.39(1.11–1.72)	1.42(1.14–1.76)
Secondary/higher		1 (reference)	1 (reference)	1 (reference)
Wealth index				
Poor		1 (reference)	1 (reference)	1 (reference)
Middle		0.57(0.43–0.75)	0.72(0.52–0.99)	0.76(0.55–1.03)
Rich		0.29(0.20–0.39)	0.44(0.31–0.64)	0.48(0.32–0.69)
Marital status				
Never married		1 (reference)	1 (reference)	1 (reference)
Ever married		1.37(0.94–2.08)	1.30(0.83–1.89)	1.38(0.77–2.27)
Maternal occupation				
Not working		1 (reference)	1 (reference)	1 (reference)
Working		0.93(0.77–1.14)	1.02(0.83–1.22)	1.02(0.83–1.23)
Sex of child				
Male (vs female)		0.93(0.78–1.09)	0.92(0.78–1.08)	0.92(0.78–1.09)
Birth order				
1st -3rd order		1 (reference)	1 (reference)	1 (reference)
4th -6th order		1.51(1.24–1.85)	1.56(1.27–1.90)	1.53(1.24–1.86)
7th + order		1.49(1.07–2.03)	1.50(1.06–2.07)	1.46(1.02–2.02)
Size of child at birth				
Large		1 (reference)	1 (reference)	1 (reference)
Average		1.11(0.92–1.32)	1.10(0.93–1.29)	1.13(0.94–1.35)
Small		1.30(0.97–1.68)	1.28(0.98–1.64)	1.32(1.002–1.72)
Exposure to media				
Never exposed		1 (reference)	1 (reference)	1 (reference)
Exposed		0.92(0.75–1.14)	0.99(0.79–1.24)	1.03(0.79–1.31)
Antenatal care				
Never attended		1 (reference)	1 (reference)	1 (reference)
Attended		0.45(0.37–0.54)	0.48(0.38–0.58)	0.49(0.39–0.60)
Place of delivery				
Home		1 (reference)	1 (reference)	1 (reference)
Health facility		0.57(0.46–0.69)	0.61(0.49–0.75)	0.62(0.51–0.74)
Community-level factors				
Residence				
Urban(vs Rural)			0.67(0.52–0.84)	0.66(0.50–0.82)
Getting to health facility				
Not a problem			1 (reference)	1 (reference)
A problem			1.25(0.99–1.54)	1.28(1.02–1.57)
Ethnicity diversity index			0.99(0.95–1.03)	0.99(0.95–1.03)
Socioeconomic disadvantage				
Tertile 1(least disadvantaged)			1 (reference)	1 (reference)
Tertile 2			1.53(1.17–2.02)	1.44(1.09–1.88)
Tertile 3 (most disadvantaged)			3.69(2.09–6.50)	2.93(1.60–4.71)
State-level factors				
Socioeconomic disadvantage				
Tertile 1(least disadvantaged)				1 (reference)
Tertile 2				0.88(0.57–1.28)
Tertile 3 (most disadvantaged)				2.69(1.37–4.73)
Measures of variation				
State level				
Variance (SE)	2.270(1.330–3.730)	0.388(0.179–0.740)	0.282(0.113–0.567)	0.205(0.081–0.412)
Explained variation (%)	Reference	82.9	87.6	91.0
ICC (%)	31.80	8.76	6.36	4.72
MOR	4.18	1.81	1.66	1.54
Community level				
Variance (SE)	1.578(1.216–1.979)	0.752(0.512–1.013)	0.863(0.610–1.144)	0.838(0.598–1.104)
Explained variation (%)	Reference	52.3	45.3	46.9
ICC (%)	53.91	25.74	25.82	24.06
MOR	3.30	2.28	2.42	2.39
Model fit statistics				
Bayesian DIC	4791	4581	4542	4540

*Abbreviations*: *SE* standard error, *DIC* deviation information criterion, *CrI* credible interval, *ICC* intra-cluster correlation, *MOR* median odds ratio

^a^Model 1 is the empty model, a baseline model with no independent variable

^b^Model 2 is adjusted for age, education, wealth status of family, marital status, occupation, sex of child, birth order, size of child at birth, exposure to media, antenatal care and place of delivery

^c^Model 3 is additionally adjusted for residency, getting to health facility, ethnicity diversity index and community socioeconomic factors

^d^Model 4 is additionally adjusted for state-level socioeconomic factors

### Measures of variation

There was a significant variation in the odds of not having children fully immunized across the states (*σ* ^2^_=_ 2.270; 95% CrI = 1.330–3.730) and across the communities (*σ* ^2^_=_ 1.578; 95% CrI = 1.216–1.979) as indicated in Table 2. The intra-state and intra-community correlation coefficients show that 31.8% and 53.9% of the variance in odds of not having a child fully immunized are linked to state- and community-level factors respectively. The MOR underscores the important role community- and state-level factors play in influencing child immunization status. As estimated in model 4, if a mother moved to another state or community that has a higher probability of incomplete child immunization, the likelihood of having her child not fully immunized would increase by 1.54 and 2.39 times respectively.

## Discussion

We found that individual characteristics and community and state factors are important in explaining the variations in incomplete immunization status of children in Nigeria. Children of young women (15–24 years) are more likely to be incompletely immunized when compared with children of older women. This is in agreement with previous studies [23, 24]. This implies that the probability of having a child immunized increases with increasing age of mothers. This scenario may be attributed to the child care experience which the young mothers are yet to acquire. Older mothers have gone through the rigours of caring for sick children with the resultant effects on time and household income. Such mothers would appreciate any initiative designed to prevent the occurrence of childhood illness. Our study shows the role of education in predicting child immunization. As mothers’ level of education increased, the probability of having a child not fully immunized reduced such that children of mothers with secondary or higher education have the least likelihood of not being fully immunized when compared with children whose mothers have primary or no education. Education has been reported [25–27] to have a profound effect on mother’s health seeking behaviour which includes child immunization. As revealed in similar studies [13, 28], childhood immunization is influenced by the household poverty. The poorer a household becomes, the more the tendency of children from such households to be incompletely immunized. Lack of money results in poor health seeking behaviour and this culminates into double deprivations for the children; they are deprived of nutritious food that could naturally build their immune system and they are also deprived of full immunization that could fortify them against vaccine preventable diseases.

Our findings also show that the use of health service by pregnant mothers contributes to the improvement in the immunization status of children. Children of women that attended antenatal care have lower probability of not being fully immunized compared to children whose mothers did not attend any antenatal care. This is also the view of earlier studies [29–31]. Attending antenatal care puts women in a better position of obtaining adequate information about routine immunization for children. Also, the processes undergone during antenatal care prepare a mother towards having positive inclination to health care utilization not only for themselves but also their children. Consistent with other studies [32, 33], health facility delivery has a significant effect on child immunization. Children of mothers who delivered in health facility are less likely to be incompletely immunized. Health facility delivery affords women the opportunity to have their children immunized at birth and also obtain information on subsequent immunization schedule.

Child characteristics have also been shown to influence immunization. The likelihood of getting a child fully immunized reduced as birth order increased. Children of higher birth order are more likely to be incompletely immunized when compared with children of lower birth order [34, 35]. This could be attributed to mothers’ diminished interest in immunization uptake for higher order children. Also, children who were small at birth have higher probability of not receiving full immunization. Findings from previous studies [36] have shown that children in this category are presented for immunization late. This may be as a result of the fragility belief in respect of small children. Parents of such children may consider them too fragile for immunization.

The effects of community- and state- level factors are highlighted in the study. Our findings indicate that the chances of not being fully immunized are higher for children in rural than urban areas [37, 38]. There are many health facilities in the urban areas and women in such areas have access to them. At the same time, information about child health is accessed through different outlets in urban areas. Mothers’ experience in getting to health facility was significantly associated with child immunization. As emphasised in earlier studies [10, 39, 40], children of mothers who experienced difficulty in reaching health facilities are more likely to be incompletely immunized. Difficulty in getting to health facilities serves as a major barrier to child immunization uptake. This is typical of those living in remote areas. The likelihood of not being fully immunized increased for children whose mothers live in socioeconomically disadvantaged communities and states. This confirms the findings of previous studies [12, 41] which revealed that socioeconomic nature of the communities influences the health seeking behaviour of individuals.

### Policy implications

Nigeria is one of the 10 countries in the world where most of the incompletely immunized children live despite the huge investments in immunization programme by governmental and non-governmental organizations. There is a disparity in the rate of immunization incompleteness between the north and south of the country with the former having a higher proportion. This disparity could be explained by the factors that operate at individual, community and state levels. Low level of educational attainment, high level of poverty, poor antenatal care attendance and hospital delivery, higher population of rural inhabitants including poor socioeconomic status are more pronounced in the northern region. Evidence from other studies also emphasized that child immunization incompleteness in the north contributes significantly to the overall low immunization uptake in Nigeria. Some of the factors attributed to this include misconception about immunization (a situation where it is believed that once a child receives polio vaccine, such child has been immunized against all childhood diseases), rejection of routine immunization (this is linked to the fear on the part of parents and care givers that vaccines are harmful to the child) and political problems (this relates to lack of commitment from the government about the immunization policy and too much centralization of the immunization programme) [42]. In view of this, our study suggests some policy measures for addressing this situation. Health service providers in conjunction with community leaders need to organize awareness programmes on regular basis at the community level. Such programmes should focus on emphasizing the importance of health service utilization for mothers and children. In addition, issues relating to misconception about vaccines and unwillingness of mothers to present children who were small at birth and those of higher birth order for immunization should be adequately discussed. While government at federal level should address the problem of over-centralization of the immunization policy, government at both state and federal levels should provide funds for the establishment of mobile clinics in order to ensure that immunization services are available to children whose mothers reside in remote areas. This would also eliminate the problem associated with lack of money for transportation. Adult education programme should be resuscitated in Nigeria to give illiterate mothers the opportunity of being educated. Government at all levels need to commit more resources to improving people’s socioeconomic status through the provision of employment opportunities, interest-free loans for small and medium scale enterprises and unemployment benefits. This would go a long way in reducing inequalities in the country.

### Study strengths and weaknesses

Some limitations of the present study should be noted. First, our findings depend on the quality of DHS data. DHS is the largest program for the collection of quantitative data on population and health from households in the developing countries and is generally considered as one of the most reliable sources of maternal and child health data. Furthermore, the participants’ survey is cross-sectional, so causal relationships between variables of interest cannot be assessed. In addition, because the data were self-reported, it is possible that the responses were affected by recall and social desirability bias.

However, the survey is population-based which covered all the states and regions in the country. This, therefore, allows the results from this study to be generalized to the studied population and other countries in sub-Saharan Africa with similar settings.

## Conclusions

This study has shown that individual-, community- and state-level factors significantly influence child immunization incompleteness in Nigeria. Interventions to improve child immunization uptake should take into consideration the individual, community and state level characteristics. It will be of great benefit if all these factors are properly considered during planning, formulation and implementation of policies by governmental and non-governmental organizations that will improve childhood immunization coverage in Nigeria and other countries.

## Abbreviations

aOR: Adjusted odds ratio
CrI: Credible interval
DIC: Deviation information criterion
ICC: Intra-Cluster Correlation
MOR: Median odds ratio
NDHS: Nigeria demographic and health survey
SE: Standard error
